# Montelukast Induces Apoptosis-Inducing Factor-Mediated Cell Death of Lung Cancer Cells

**DOI:** 10.3390/ijms18071353

**Published:** 2017-06-24

**Authors:** Ming-Ju Tsai, Wei-An Chang, Pei-Hsun Tsai, Cheng-Ying Wu, Ya-Wen Ho, Meng-Chi Yen, Yi-Shiuan Lin, Po-Lin Kuo, Ya-Ling Hsu

**Affiliations:** 1 Division of Pulmonary and Critical Care Medicine, Department of Internal Medicine, Kaohsiung Medical University Hospital, Kaohsiung Medical University, Kaohsiung 807, Taiwan; SiegfriedTsai@gmail.com (M.-J.T.); 960215kmuh@gmail.com (W.-A.C.); kanginbobo@gmail.com (P.-H.T.); 2 Department of Internal Medicine, School of Medicine, College of Medicine, Kaohsiung Medical University, Kaohsiung 807, Taiwan; 3 Graduate Institute of Clinical Medicine, College of Medicine, Kaohsiung Medical University, Kaohsiung 807, Taiwan; wu_cheng_ying@hotmail.com (C.-Y.W.); gladys505202@hotmail.com (Y.-W.H.); yohoco@gmail.com (M.-C.Y); ysirenelin@gmail.com (Y.-S.L.); 4 Department of Respiratory Therapy, School of Medicine, College of Medicine, Kaohsiung Medical University, Kaohsiung 807, Taiwan; 5 Graduate Institute of Medicine, College of Medicine, Kaohsiung Medical University, Kaohsiung 807, Taiwan; 6 Department of Emergency Medicine, Kaohsiung Medical University Hospital, Kaohsiung Medical University, Kaohsiung 807, Taiwan

**Keywords:** cysteinyl leukotriene receptor antagonists, montelukast, lung cancer, apoptosis-inducing factor, asthma

## Abstract

Developing novel chemo-prevention techniques and advancing treatment are key elements to beating lung cancer, the most common cause of cancer mortality worldwide. Our previous cohort study showed that cysteinyl leukotriene receptor antagonists, mainly montelukast, decreased the lung cancer risk in asthma patients. In the current study, we conducted in vivo and in vitro experiments to demonstrate the inhibiting effect of montelukast on lung cancer and to investigate the underlying mechanisms. Using Lewis lung carcinoma-bearing mice, we showed that feeding montelukast significantly delayed the tumor growth in mice (*p* < 0.0001). Montelukast inhibited cell proliferation and colony formation and induced the cell death of lung cancer cells. Further investigation showed the down-regulation of B-cell lymphoma 2 (Bcl-2), up-regulation of Bcl-2 homologous antagonist/killer (Bak), and nuclear translocation of apoptosis-inducing factor (AIF) in montelukast-treated lung cancer cells. Montelukast also markedly decreased the phosphorylation of several proteins, such as with no lysine 1 (WNK1), protein kinase B (Akt), extracellular signal-regulated kinase 1/2 (Erk1/2), MAPK/Erk kinase (MEK), and proline-rich Akt substrate of 40-kDa (PRAS40), which might contribute to cell death. In conclusion, montelukast induced lung cancer cell death via the nuclear translocation of AIF. This study confirmed the chemo-preventive effect of montelukast shown in our previous cohort study. The utility of montelukast in cancer prevention and treatment thus deserves further studies.

## 1. Introduction

Cancer is a leading cause of morbidity and mortality [1,2,3]. Although much advancement has been made in lung cancer treatment, lung cancer remains the most common cause of cancer death [1,2,3]. Lung cancer is usually diagnosed in the advanced stages, and drug resistance develops sooner or later during the treatment course, leading to unsatisfying outcomes. Developing cancer-preventing strategies is therefore as important as advancing anti-cancer strategies [1,2].

In our previous nationwide population-based cohort study, we found that cysteinyl leukotriene receptor antagonists (LTRAs) decreased cancer risk in a dose-dependent manner in asthma patients, and the significant chemo-preventing effect of LTRA was mainly observed in lung, breast, colorectal, and liver cancers [1]. LTRAs, such as montelukast and zafirlukast, are widely used for treating allergic asthma and rhinitis [4,5], while montelukast was about ten times more widely used than zafirlukast in asthma patients in Taiwan [1]. The leukotriene pathway, in addition to its well-known role in allergic disease, is also responsible for carcinogenesis and tumor-mediated immune-suppression [6]. Few in vitro studies have shown the anti-cancer effect of montelukast in various genital and urological cancer cells [7,8,9,10,11,12], and only a few in vivo studies have reported the anti-cancer effect of leukotriene pathway inhibitors [12,13,14,15]. However, the effect of montelukast on lung cancer has not been studied yet, and its underlying anti-cancer mechanisms have not been fully understood.

As dysregulated cell death and increased proliferation are associated with carcinogenesis, one of the most important approaches to develop novel anti-cancer treatment is discovering therapeutic agents that induce cancer cell death. Historically, cell death was classified into apoptosis and necrosis [16]. Caspase-dependent (classical) apoptosis, occurring via two canonical pathways (death-receptor-mediated and mitochondria-mediated), is a well-known form of programmed cell death [17]. Apoptosis may also occur via caspase-independent routes, and one of the key routes is mediated by the translocation of apoptosis-inducing factor (AIF) from the mitochondria to the nucleus [17]. Necrosis, characterized by the release of the cell content due to the disruption of the cell membrane, was traditionally considered passive, accidental, and uncontrolled [16,17]. In the last decade, programmed necrosis, a caspase-independent programmed cell death mainly mediated by the nuclear translocation of AIF, has been described [16,17,18,19]. Cumulating evidence has shown that AIF in the nucleus contributes to chromatin condensation, DNA fragmentation, and nuclear shrinkage, contributing to programmed cell death [16,17,18,19].

The major aim of this study was to demonstrate the inhibiting effect of montelukast on lung cancer and to investigate the underlying mechanisms. 

## 2. Results

### 2.1. Montelukast Induced Cell Death of Lung Cancer Cells

**Figure 1:**
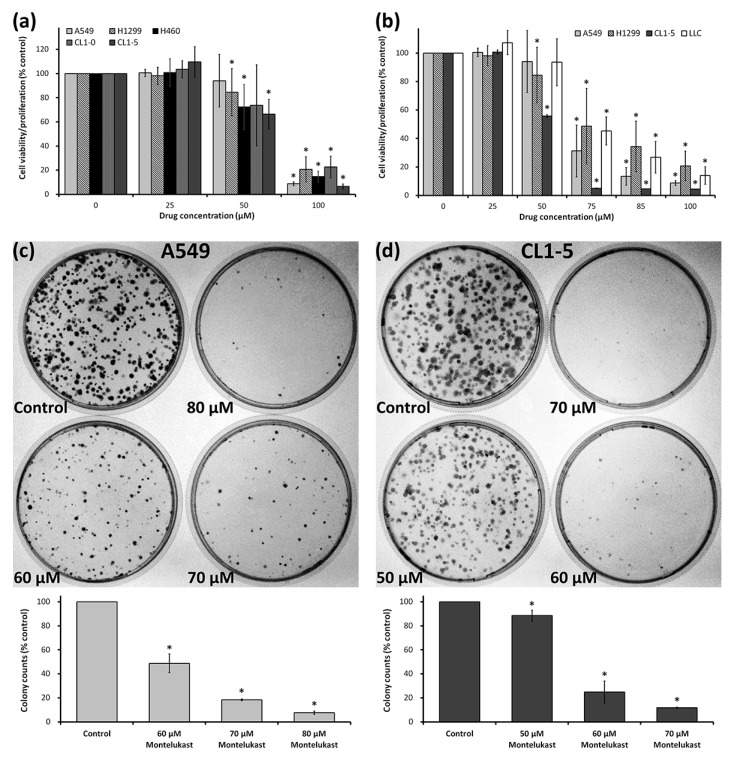
Montelukast inhibited the viability/proliferation of lung cancer cells. (**a**,**b**) The cells were treated with various concentrations of montelukast for two days. Cell proliferation/viability was determined by a Premixed Water-Soluble Tetrazolium Salt-1 (WST-1) Cell Proliferation Assay; (**c**,**d**) after being treated with various concentrations of montelukast for a day, the cells (A549 and CL1-5) were incubated for nine days and the colonies on each dish were counted. All results were expressed as the mean ± SD of independent experiments performed on different days. * *p* < 0.05, as compared with the corresponding control (0 μM) group.

**Figure 2:**
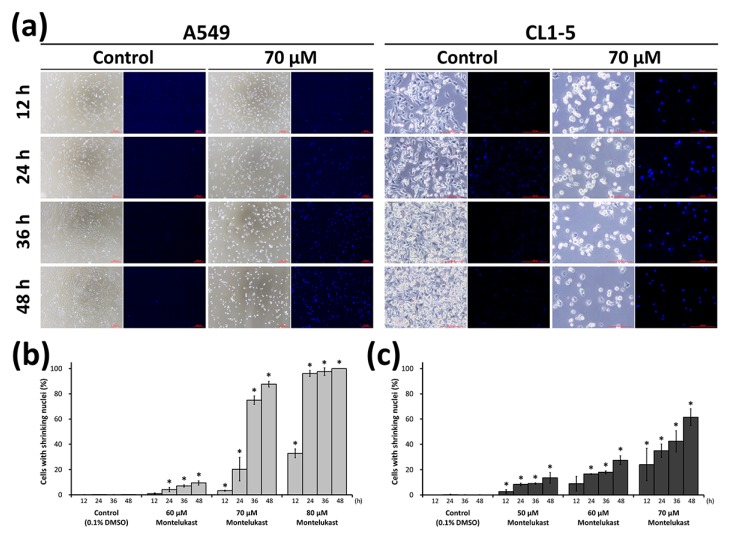
Montelukast-induced cell death of lung cancer cells. After being treated with various concentrations of montelukast for the indicated time (12, 24, 36, or 48 h), the cells (A549 and CL1-5) were observed with light microscopy and fluorescence microscopy (4’,6-diamidino-2-phenylindole (DAPI) staining). (**a**) Representative photographs of the cells were shown (The detailed photographs are presented in Figure S1); (**b**,**c**) The percentages of A549 (**b**) and CL1-5 (**c**) cells with shrinking nuclei were calculated. All results were expressed as the mean ± SD of three independent experiments performed on different days. * *p* < 0.05, as compared with the corresponding control (0 μM) group.

To further confirm the cytotoxic effect of montelukast on lung cancer cells, we treated a variety of lung cancer cell lines with various concentrations of montelukast. Using a Water-Soluble Tetrazolium Salt-1 (WST-1) Cell Proliferation Assay, we found that 100 μM of montelukast induced more than 75% of growth inhibition in A549, H1299, H460, CL1-0, CL1-5, and Lewis lung carcinoma (LLC) (Figure 1a,b). Further analysis found that the 50% inhibitory concentration of montelukast was between 50 and 75 μM in A549, H1299, CL1-5, and LLC (Figure 1b). We further carried out an anchorage-independent clonogenic assay, an established method using soft agar, to test the tumorigenic ability of cancer cells in vitro [12]. After being treated with various concentrations of montelukast, the colony-forming ability of lung cancer cells was significantly reduced in a dose-dependent manner (Figure 1c,d). We further examined the lung cancer cells treated with various concentrations of montelukast microscopically. The cells treated with montelukast showed characteristics of cell death, including cellular shrinkage, nuclear shrinkage, and chromatin condensation (Figure 2 and Figure S1). The percentage of cells with shrinking nuclei significantly increased with montelukast treatment in a dose-dependent and time-dependent manner.

### 2.2. Montelukast Induced Cell Death of Lung Cancer Cells via Nuclear Translocation of Apoptosis-Inducing Factor

**Figure 3:**
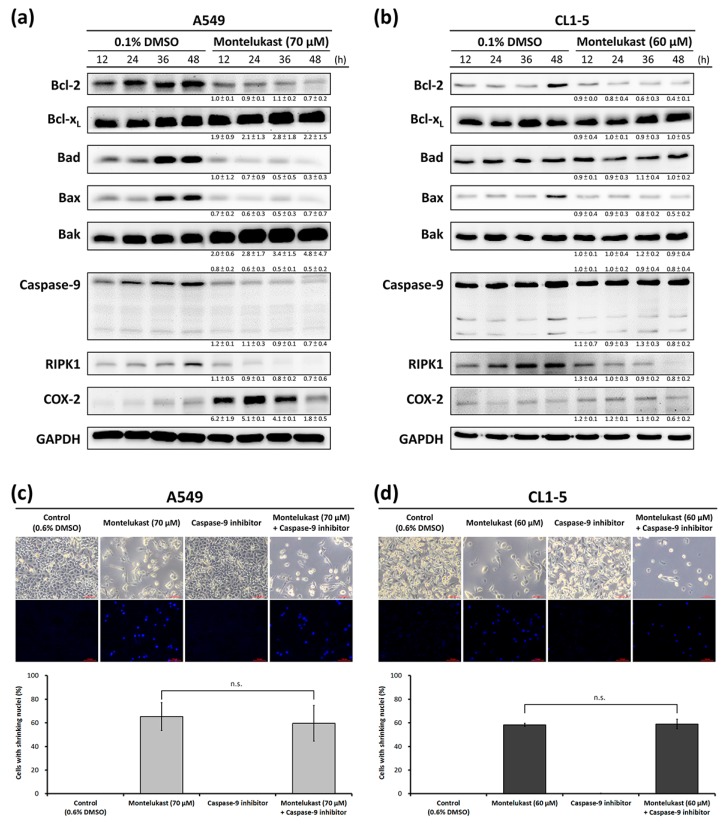
The montelukast-induced death of lung cancer cells did not depend on various proteins in the Bcl-2 family or caspase-9. (**a**,**b**) The cells (A549 and CL1-5) were treated with 0.1% dimethyl sulfoxide (DMSO) (control) or montelukast for the indicated time (12, 24, 36, or 48 h). The levels of various proteins in cell lysates were assessed with immunoblot assay. The results shown were representatives of at least three independent experiments performed on different days, along with the means ± SD of the relative expression levels to the corresponding control groups at the same time point; (**c**,**d**) the cells (A549 and CL1-5) were pre-treated with or without a specific caspase-9 inhibitor (20 μM) for 1 h, and then treated with 0.6% DMSO (control) or montelukast for 48 h. The percentages of cells with shrinking nuclei were calculated. All results were expressed as the mean ± SD of three independent experiments performed on different days. n.s., no significant difference (*p* > 0.5).

To investigate the possible mechanisms of the montelukast-induced cell death of lung cancer cells, the expression levels of apoptosis-associated proteins were analyzed with immunoblot. Montelukast treatment markedly decreased the expression of Bcl-2 and markedly increased the expression of Bak in a time-dependent manner in A549 and CL1-5 (Figure 3a,b). However, the changing trend in the expression levels of Bcl-xL, Bad, and Bax was not compatible with classical apoptosis. The expression level of caspase 9 was markedly decreased in A549, but not in CL1-5. By pretreating the cells with a specific inhibitor of caspase 9, the caspase-9-independent nature of the montelukast-induced cell death of lung cancer cells was confirmed (Figure 3c,d). In addition, the expression level of RIPK1 was markedly decreased in montelukast-treated cells, excluding the participation of necroptosis in montelukast-induced cell death (Figure 3a,b). Interestingly, the expression level of cyclooxygenase-2 (COX-2) was markedly increased in montelukast-treated A549 cells (Figure 3a,b).

**Figure 4:**
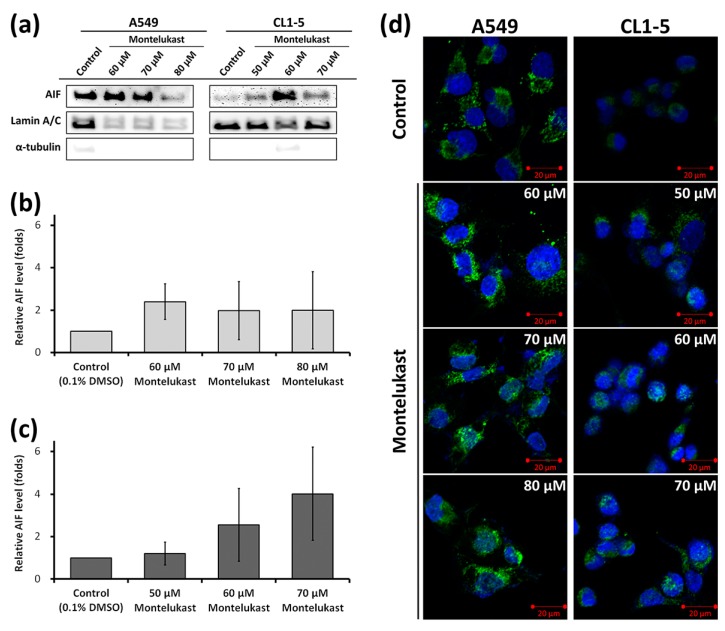
Montelukast-induced nuclear translocation of apoptosis-inducing factor (AIF) in lung cancer cells. (**a**–**c**) The cells (A549 and CL1-5) were treated with 0.1% dimethyl sulfoxide (DMSO) (control) or montelukast for 24 h. The levels of AIF in the nuclei were assessed with immunoblot assay. The results shown are representative photographs (**a**) of multiple experiments, along with the means ± SD of the relative levels to the lamin A/C levels and to the control groups (**b**, A549; **c**, CL1-5); (**d**) The cells (A549 and CL1-5) were treated with 0.1% DMSO (control) or various concentrations of montelukast for 24 h. The nuclear translocation of AIF was demonstrated with immunofluorescence staining and laser scanning confocal microscopy. Blue, DAPI-labeled nuclei; green, AIF.

To investigate whether apoptosis-inducing factor (AIF) participates in montelukast-induced cell death, its levels in the nuclei were assessed. Montelukast markedly increased the levels of AIF in the nuclear fragments (Figure 4a–c). Using confocal microscopy, the nuclear translocation of AIF induced by montelukast treatment was clearly demonstrated (Figure 4d).

### 2.3. Montelukast Decreased Phosphorylation of With No Lysine 1 (WNK1), Protein Kinase B (Akt), Extracellular Signal-Regulated Kinase 1/2 (Erk1/2), MAPK/Erk kinase (MEK), and Proline-Rich Akt Substrate of 40-kDa (PRAS40) in Lung Cancer Cells

**Figure 5:**
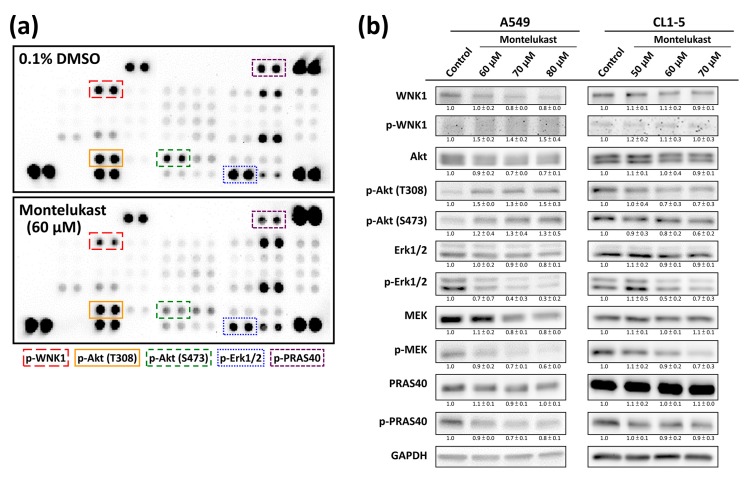
Montelukast decreased the phosphorylation of With No Lysine 1 (WNK1), protein kinase B (Akt), Extracellular Signal-Regulated Kinase 1/2 (Erk1/2), MAPK/Erk kinase (MEK), and Proline-Rich Akt Substrate of 40-kDa (PRAS40) in lung cancer cells. (**a**) The CL1-5 cells were treated with 0.1% DMSO (control) or 60 μM montelukast for 8 h. The levels of protein phosphorylation in the cell lysates were assessed with a Human Phospho-Kinase Array Kit; (**b**) The cells (A549 and CL1-5) were treated with 0.1% DMSO (control) or various concentrations of montelukast for 8 h. The phosphorylated and total protein levels in the cell lysates were assessed with an immunoblot assay. The results shown are representatives of at least three independent experiments performed on different days, along with the means ± SD of the relative expression levels to the corresponding control groups at the same time point.

We screened the protein phosphorylation pattern with a Human Phospho-Kinase Array Kit to investigate the possible regulator of montelukast-induced cell death and found a markedly decreased phosphorylation of WNK1, Akt, Erk1/2, MEK, and PRAS40 in montelukast-treated CL1-5 (Figure 5a). Using an immunoblot assay (Figure 5b), we confirmed these findings in CL1-5 cells, whereas the phosphorylation of WNK1 and Akt was markedly increased in A549 cells.

### 2.4. Montelukast Inhibited Tumor Growth in Mice

**Figure 6:**
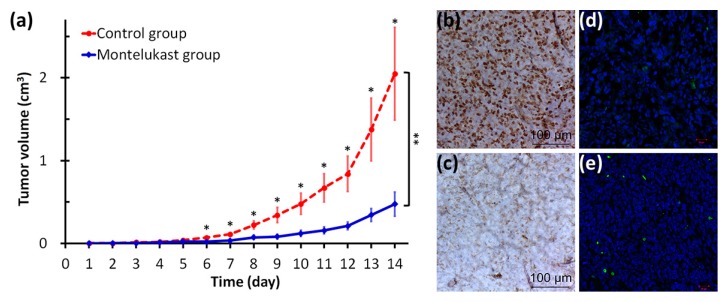
In vivo study showing the anticancer effect of montelukast. The C57BL/6JNarl (B6) mice were given either water (control group) or 100 μg of montelukast in water (treatment group) via tube feeding daily from day −3 to day 13. Each mouse was injected with 500,000 Lewis lung carcinoma (LLC) cells subcutaneously on day 0. The mice were sacrificed on day 14 and the tumor specimens were sent for a pathological exam. (**a**) The tumor volume was shown in mean (±standard error of mean) for each group on each day (from day 1 to day 14) and plotted. * *p* < 0.05, while comparing the tumor volumes in both groups on the same day. ** We adapted a mixed effect model based on the tumor volume with the effects of group, time, and their interaction, while taking into account the repeated observations of study mice. All three effects showed significantly different tumor growth [group (montelukast group vs. control group), *p* = 0.0369; time (in days), *p* < 0.0001; their interaction (growth rates of montelukast group vs. control group), *p* < 0.0001]; (**b**,**c**) immunohistochemical staining showed a markedly decreased Ki-67 expression in the tumor specimens from the montelukast group (**c**) compared to the control group (**b**); (**d**,**e**) confocal microscopy of the terminal deoxynucleotidyl transferase dUTP nick end labeling (TUNEL) assay showed a markedly increased number of TUNEL-positive cells in the tumor specimens from the montelukast group (**e**) compared to the control group (**d**).

To confirm the chemo-preventive effect of cysteinyl leukotriene receptor antagonists shown in our previous study [1], as well as to confirm our in vitro findings, we arranged an animal study using cancer-bearing mice. Mice with Lewis lung carcinoma (LLC) cells injected subcutaneously were given either water or montelukast daily from the third day prior to the LLC cells injection. The mice fed with montelukast had a significantly slower tumor growth rate than the control group (*p* < 0.0001) (Figure 6a). The tumor specimens collected from the montelukast group showed a markedly decreased Ki-67 expression (Figure 6b,c) and increased terminal deoxynucleotidyl transferase dUTP nick end labeling (TUNEL)-positive cells (Figure 6d,e) than those collected from the control group.

## 3. Discussion

**Figure 7:**
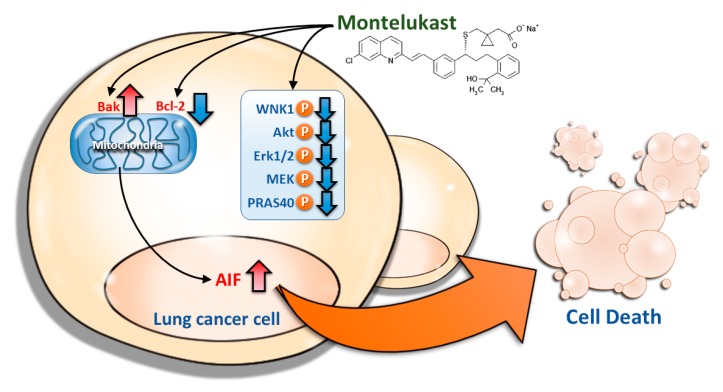
Illustration of the mechanism through which montelukast induced the cell death of lung cancer cells. Montelukast decreased the phosphorylation of several proteins, such as WNK1, Akt, Erk1/2, MEK, and PRAS40, down-regulated Bcl-2, up-regulated Bak, and induced the nuclear translocation of AIF, contributing to the cell death of lung cancer cells. (Red upward arrows: up-regulation; blue downward arrows: down-regulation)

In our previous study analyzing the Taiwan National Health Insurance Database, we demonstrated that LTRAs, mainly montelukast, decreased cancer risk in a dose-dependent manner in asthma patients, and the cancer risk reduction was mainly observed in lung, breast, colorectal, and liver cancers [1]. The findings from the cohort study was confirmed in the present study using LLC-bearing mice to showed the chemo-preventive effect of montelukast. Further investigation showed that montelukast induced lung cancer cell death via the nuclear translocation of AIF (Figure 7). Montelukast also markedly decreased the phosphorylation of several proteins, such as WNK1, Akt, Erk1/2, MEK, and PRAS40, which might contribute to cell death (Figure 7).

Inflammatory immune responses are crucial mediating mechanisms in the tumor microenvironment [20]. The cross-talk between cancer cells and the surrounding cells in the microenvironment, especially the immune cells, contributes to the formation of a milieu suitable for carcinogenesis and cancer progression [20,21]. Eicosanoids, metabolized from arachidonic acid by cyclooxygenase (COX), lipoxygenase (LOX), or the p450 epoxygenase pathway, are involved in various immune responses, and are important inflammatory mediators in the tumor microenvironment [6,20,22]. COX-derived prostanoids contribute to the pathogenesis of cancer, and blocking the COX pathway with nonselective non-steroidal anti-inflammatory drugs or selective COX-2 inhibitors may reduce cancer risks [6,23,24,25]. In recent years, a number of studies have indicated the important role of LOX-derived leukotrienes in carcinogenesis and cancer progression [6,20,26]. The tumor-promoting role of the LTB_4_ pathway has been recognized in many cancers, such as prostatic cancer [27], esophageal cancer [28], head and neck cancer [29], gastric cancer [30], and colon cancer [31]. Cysteinyl leukotrienes (LT), including LTC_4_, LTD_4_, and LTE_4_, which are originally recognized for their effects in promoting bronchoconstriction, inflammation, mucus secretion, and microvascular permeability, have also been gradually understood for their contribution in carcinogenesis and cancer progression [4,6,20]. LTD_4_, mainly through the stimulation of its principle receptor, cysteinyl leukotriene receptor 1 (CysLT_1_R), promotes cell survival, proliferation, migration, and the transcription of potentially oncogenic genes [25]. Patients with hepatocellular carcinoma had a significantly higher circulating LTD_4_ level than healthy subjects [32]. LTD_4_ treatment augmented the growth of the xenograft tumor (using a colon cancer cell line, HCT-116) in nude mice [33]. A recent study using mice with a global disruption of the cysteinyl leukotriene receptor gene (*Cysltr1*) also demonstrated the important role of CysLT_1_R in colon carcinogenesis [34]. Clinically, an increased expression of CysLT_1_R was noted in specimens from gastric cancer [30], renal cell carcinoma [7,8], transitional cell carcinoma [7,9], prostate cancer [7,10], and testicular cancer [7,11]. Furthermore, the elevated CysLT_1_R was correlated to poor prognosis in patients of breast cancer [35] and colon cancer [36,37]. 

Some in vitro studies have demonstrated the overexpression of CysLT_1_R in various cancer cells, including renal cell carcinoma [7,8], transitional cell carcinoma [7,9], prostate cancer [7,10], testicular cancer [7,11], and colon cancer [12], while montelukast induced the apoptosis of these cancer cells. A study of human neuroblastoma demonstrated that montelukast inhibited cancer cell growth by inducing cell cycle arrest at the G_1_ phase and apoptosis [38]. Another study using HCT-116, a colon cancer cell line, also showed that montelukast inhibited cell proliferation, cell adhesion, and colony formation, as well as induced cell cycle arrest at the G_1_ phase and apoptosis of the cancer cells [12]. In line with these studies, the present study showed that montelukast inhibited cell proliferation and colony formation, and induced lung cancer cell death.

Only a few in vivo studies in the literature to date have shown the chemo-preventive effect of leukotriene pathway inhibitors [12,13,14,15]. An early study using a mouse model of vinyl carbamate-inducing lung tumors demonstrated the chemo-preventive effect of leukotriene pathway inhibitors, including accolate (zafirlukast), zileuton, and MK-866 [13]. A recent study using a mouse model of *n*-nitroso *n*-methyl urea-inducing mammary carcinogenesis also demonstrated the chemo-preventive effect of montelukast [15]. In a study using a peripheral spontaneous metastasis model, LLC cells were inoculated into the foot pad of mice; after the tumor-inoculated foot was amputated, mice given pranlukast or montelukast had a significantly prolonged survival than the mice in the control group [14]. A recent study using nude mice with colon cancer xenografts demonstrated that LTRAs, ZM198,615, and montelukast, inhibited proliferation and increased the apoptosis of the tumor cells inoculated subcutaneously [12]. In line with these studies, our present study showed significantly delayed tumor growth, with inhibited proliferation and increased apoptosis, of the LLC cells inoculated subcutaneously in the mice.

The mechanisms through which montelukast induces cancer cell death have not yet been well studied. As shown in a study using human neuroblastoma cells, montelukast induced the cleavage of caspase 9 and caspase 3 [38]. The study using colon cancer cells demonstrated an increased p21 level and cleaved caspase 3 in montelukast-treated cells [12]. Using caspase-9 inhibitor in the current study, the participation of caspase 9 was excluded from montelukast-induced lung cancer cell death. The mitochondrial membrane integrity is regulated by Bcl-2 family proteins. Bcl-2 stabilizes the mitochondrial membrane barrier function, sequestrating apoptogenic proteins in the mitochondria; Bak permeabilizes the mitochondrial membrane [39,40]. The nuclear translocation of AIF, released from the mitochondria, is a critical event contributing to caspase-independent programmed cell death [16,17,18,19,39,40,41,42]. The findings in our current study suggested that montelukast down-regulated Bcl-2 and up-regulated Bak, causing the mitochondrio-nuclear translocation of AIF, resulting in cell death.

Many signaling pathways involved in cell survival and proliferation are usually dysregulated in cancer cells. Mitogen-activated protein kinase (MAPK) cascades, especially those involving extracellular signal-regulated kinase (Erk) 1/2 activated by MAPK/Erk kinase (MEK) 1/2 dual-specificity protein kinases, promote cancer cell survival and migration [21,43]. In line with the studies showing the role of the LTD_4_-induced activation of MEK1/2 and Erk1/2 in cell proliferation [44,45], the present study found that montelukast decreased Erk1/2 phosphorylation, which might contribute to the montelukast-induced cell death. As the proline-rich Akt substrate of the 40-kDa (PRAS40) protein may protect the cells against apoptosis through its direct inhibiting effect on the mammalian target of rapamycin (mTOR) pathway [46], the montelukast-induced decreased PRAS40 phosphorylation might also contribute to montelukast-induced cell death. With no lysine 1 (WNK1), which may be activated by Akt, is important in cell proliferation and invasion, contributing to carcinogenesis and cancer progression [21,47,48,49]. Therefore, the decreased phosphorylation of WNK1 and Akt in CL1-5 cells treated with montelukast might contribute to the montelukast-induced cell death. Interestingly, the phosphorylation of WNK1 and Akt was markedly increased in A549 cells treated with montelukast, and this might provide an explanation for the higher dose of montelukast needed to achieve a similar extent of cell death in A549 cells as in CL1-5 cells.

Most previous studies showed that LTD_4_ might induce the up-regulated expression and activation of COX-2 via the activation of Erk1/2 [25,44]. Interestingly, our present study found that montelukast induced the up-regulation of COX-2 in A549 lung cancer cells. This shunting toward the COX pathway might be an escape mechanism in the cancer cells to counteract montelukast-induced apoptosis [20]. This might also provide an explanation for the higher dose of montelukast required to induce a similar extent of cell death in A549 cells as in CL1-5 cells. Indeed, a combined inhibition of the COX and LOX pathways simultaneously has been proposed. In a study using a colon cancer-bearing nude mice xenograft model, combined treatment with COX-2 and 5-LOX inhibitors, resulted in better tumor growth inhibition than using a single agent alone [20,50]. Besides, montelukast was useful to prevent the adverse reactions related to excessive leukotriene production while using COX-2 inhibitors [51]. Therefore, combining montelukast with a COX-2 inhibitor might be considered for developing a novel anti-cancer or chemo-preventive regimen.

The dosing of current anti-cancer drugs is usually limited by the potential side effects, leading to less treatment effects. How to make the most of the current anti-cancer drugs remains an important issue. Combining few drugs in lower doses, adding adjuvant agents, and selecting a responsive population are common strategies. For example, our previous study showed that C-X-C motif chemokine 5 (CXCL5) antibody synergistically enhanced the effect, without increasing the toxicity, of gefitinib, an epidermal growth factor receptor tyrosine kinase inhibitor, in treating lung cancer [21]. Given its great safety profiles, we believe that montelukast might also be used as an adjuvant to current anti-cancer treatment.

## 4. Materials and Methods 

### 4.1. Cell Culture

Human lung cancer cells, including A549, H460, and H1299, were obtained from the American Type Culture Collection (ATCC, Manassas, VA, USA). Less invasive (CL1-0) and highly invasive (CL1-5) human lung adenocarcinoma cell lines were generously provided by Prof. Pan-Chyr Yang (Department of Internal Medicine, National Taiwan University Hospital, Taipei, Taiwan). A549 was cultured in modified Eagle’s medium (MEM) (Lonza, Walkersville, MD, USA) supplemented with 10% fetal bovine serum (FBS), sodium pyruvate, L-glutamine, 1% non-essential amino acids, and 1% penicillin-streptomycin (Lonza, Walkersville, MD, USA). H460, H1299, CL1-0, and CL1-5 were cultured in Roswell Park Memorial Institute (RPMI) 1640 medium supplemented with 10% FBS and 1% penicillin-streptomycin (Lonza, Walkersville, MD, USA). 

Mouse Lewis lung carcinoma cell line, LLC1 (LL/2), was obtained from ATCC and was maintained in Dulbecco’s modified Eagle’s medium (DMEM) (Lonza, Walkersville, MD, USA), which was supplemented with 10% FBS and penicillin/streptomycin (100 U/0.1 mg/mL) (Life Technologies, Grand Island, NY, USA). 

### 4.2. Chemicals

Montelukast was purchased from Sigma-Aldrich Co. (St. Louis, MO, USA). Reagents were dissolved in dimethyl sulfoxide (DMSO) (Sigma Chemical Co., St. Louis, MO, USA). Control cultures received the carrier solvent (0.1% DMSO), unless otherwise specified. Antibodies were purchased from Cell Signaling Technology, Inc. (Beverly, MA, USA) and Millipore Co. (Billerica, MA, USA). All other chemicals used were of the purest form available commercially.

### 4.3. Cell Proliferation Assay

Using a Premixed WST-1 Cell Proliferation Assay (Takara Bio Inc., Shiga, Japan), cell proliferation/viability was determined. In brief, the cells were incubated with the reagent for 0.5–4 h. In viable and metabolically active cells, the tetrazolium salt WST-1 was cleaved to a formazan-class dye by mitochondrial succinate-tetrazolium reductase [52]. Therefore, quantitating the formazan dye by measuring the absorbance at 450 nm in a multi-well plate reader (FLx80, BioTek, Winooski, VT, USA) provided a measurement of cell proliferation and viability.

### 4.4. Clonogenic Assay

To determine the long-term effect of montelukast, 1000 cells were seeded in a 6-cm dish and incubated overnight to attach. Cells were treated with montelukast or vehicle control for 24 h and then incubated in fresh medium for nine days (medium was replaced every three to four days). Colonies were stained with crystal violet (0.4 g/L; Sigma, St. Louis, MO, USA) and the number of colonies was counted.

### 4.5. Immunofluorescence Analysis

The cells were treated with montelukast or vehicle control. Followed by two times of washing with ice-cooled PBS, the cells were then fixed in 4% paraformaldehyde for 10 min. After being stained with 4′,6-diamidino-2-phenylindole (DAPI) for 5 min and washed with PBS for two times, they were examined under Leica DM IL LED with a DC100 Digital Camera (Leica, Wetzlar, Germany). For each slide, at least two representative low-power fields were captured (both light microscopy and fluorescence microscopy) and the percentages of cells with shrinking nuclei were calculated.

### 4.6. Immunoblot Assay

The cells were lysed on ice for 60 min by RIPA lysis buffer (0.5 M Tris-HCl, pH 7.4, 1.5 M NaCl, 2.5% deoxycholic acid, 10% NP-40, 10 mM EDTA) (Millipore Corporation, Billerica, MA, USA). After the cell lysate was centrifuged at 14,000× *g* for 15 min, the supernatant fraction was collected for immunoblot. Equivalent amounts of protein were resolved by SDS-PAGE (8–12%) and transferred to polyvinylidene difluoride membranes. After blocking for 1 h in 5% non-fat dry milk in tris-buffered saline, the membrane was incubated with the desired primary antibody for 1–16 h. After being treated with the appropriate peroxidase-conjugated secondary antibody, the immune-reactive proteins were detected using an enhanced chemiluminescence kit (Millipore, Bedford, MA, USA) according to the manufacturer’s instructions. Each membrane was also divided or stripped to examine the level of loading control (GAPDH or lamin A/C).

Using ImageJ (National Institutes of Health; available online: https://imagej.nih.gov/ij/), the gels were analyzed with densitometry. The average values of multiple replicates, which were normalized to the corresponding internal controls, are presented along with the representative figures.

### 4.7. Subcellular Fractionation

Subcellular fractionation was done using a Nuclear Extract kit (Active Motif Europe, Rixensart, Belgium) according to the manufacturers’ instructions.

### 4.8. Immunofluorescence Confocal Microscopy

To demonstrate the nuclear translocation of apoptosis-inducing factor (AIF), 70,000 cells were seeded in each well of the Millicell EZ Slides (Merck Millipore, Billerica, MA, USA) and incubated overnight to attach. Cells were treated with montelukast or vehicle control for 24 h, followed by two times of washing with ice-cooled phosphate-buffered saline (PBS). The cells were then fixed in 4% paraformaldehyde for 15 min, permeabilized with 0.25% Triton X-100 for 15 min, and then blocked in PBS supplemented with 5% bovine serum albumin (BSA) overnight. After washing twice with 1% BSA in PBS (1% BSA-PBS), the cells were probed for AIF with anti-AIF antibody (1:100) overnight at 4 °C. After washing with 1% BSA-PBS, slides were incubated with Alexa Fluor^®^ 488 goat anti-rabbit antibody (Life Technologies, Eugene, OR, USA) (1:200) in the dark at room temperature for 1 h, followed by incubation with DAPI (1:1000) in the dark at room temperature for 5 min. Slides were sealed with coverslips and then examined under a Zeiss LSM 700 confocal microscope (Carl Zeiss Ltd., Jena, Germany).

### 4.9. Human Phosphor-Kinase Array

The cells were treated with 0.1% DMSO (control) or 60 μM montelukast for 8 h. The levels of protein phosphorylation in the cell lysates were assessed with a Human Phospho-Kinase Array Kit (R&D Systems, Inc., Minneapolis, MN, USA) according to the manufacturers’ instructions.

### 4.10. Animal Study

The C57BL/6JNarl (B6) (four to six weeks old) were purchased from the National Laboratory Animal Center (Taipei, Taiwan) and maintained in a specific pathogen-free condition in the Experimental Animal Center under the supervision of board-certified veterinarians. After adapting for a week in the Experimental Animal Center, the mice were used for experiments. All animal experiments followed the protocol approved by the Institutional Animal Care and Use Committee (IACUC) (Kaohsiung Medical University IACUC approval number: 103125).

The mice were given either water (control group) or 100 μg of montelukast in water (treatment group) via tube feeding daily from day −3 to day 13. Each mouse was injected with 500,000 LLC cells subcutaneously on day 0. In order to determine the tumor volume by an external caliper, the greatest longitudinal diameter (length) and the greatest transverse diameter (width) were determined; the subcutaneous tumor volume based on caliper measurements was calculated by the modified ellipsoidal formula (tumor volume = 0.5 × (length × width × width)) [53]. To investigate the effect of montelukast on tumor growth, we adapted a mixed effect model based on the tumor volume with the effects of group, time, and their interaction, while taking into account the repeated observations of study mice. The mice were sacrificed while animal suffering was detected or the tumor volume exceeded the preset humane endpoint, and the tumor specimens were collected for pathological examinations, including the immune-histochemical staining of Ki-67 and confocal microscopy of a TUNEL assay.

### 4.11. Statistical Analysis

Data were expressed as mean values with standard deviations (means ± SD) unless otherwise indicated. Statistical comparisons of the results were made using analysis of variance (ANOVA). All comparisons were two-tailed, and *p*-values less than 0.05 were considered significant. The extraction and computation of data, data linkage, processing and sampling, and statistical analyses were performed using SAS system (version 9.4 for Windows, SAS Institute Inc., Cary, NC, USA). The statistical significance level was set at a two-sided *p* value of <0.05.

## 5. Conclusions

Montelukast induced lung cancer cell death via the nuclear translocation of AIF. This study confirmed the chemo-preventive effect of montelukast shown in our previous study [1], as well as suggested the molecular basis of the effect. Due to its good safety profiles, montelukast deserves further study to define its role as a cancer-preventing agent, as well as to be used as an adjuvant to standard anti-cancer treatment.

## Supplementary Materials

Supplementary materials can be found at www.mdpi.com/1422-0067/18/7/1353/s1.

## Abbreviations

Akt	protein kinase B
AIF	apoptosis-inducing factor
Bak	Bcl-2 homologous antagonist/killer
Bcl-2	B-cell lymphoma 2
BSA	bovine serum albumin
COX	cyclooxygenase
CXCL5	C-X-C motif chemokine 5
CysLT1R	cysteinyl leukotriene receptor 1
DAPI	4’,6-diamidino-2-phenylindole
DMEM	Dulbecco’s modified Eagle’s medium
DMSO	dimethyl sulfoxide
EndoG	endonuclease G
Erk	extracellular signal-regulated kinase
FBS	fetal bovine serum
LLC	Lewis lung carcinoma
LOX	lipoxygenase
LT	cysteinyl leukotriene
LTRA	cysteinyl leukotriene receptor antagonist
MAPK	mitogen-activated protein kinase
MEK	MAPK/Erk kinase
MEM	modified Eagle’s medium
mTOR	mammalian target of rapamycin
PBS	phosphate-buffered saline
PRAS40	proline-rich Akt substrate of 40-kDa
TUNEL	terminal deoxynucleotidyl transferase dUTP nick end labeling
WNK1	with no lysine 1
WST-1	water-soluble tetrazolium salt-1
