# Supplementary material for: Montelukast Induces Apoptosis-Inducing Factor-Mediated Cell Death of Lung Cancer Cells
Source: Int J Mol Sci. 2017 Jun 24;18(7):1353. doi: 10.3390/ijms18071353 (PMC5535846; doi:10.3390/ijms18071353)
Supplement: Supplementary file 1 [file ijms-18-01353-s001.pdf]

# Supplementary Material: Montelukast Induces Apoptosis-Inducing Factor-Mediated Cell Death of Lung Cancer Cells

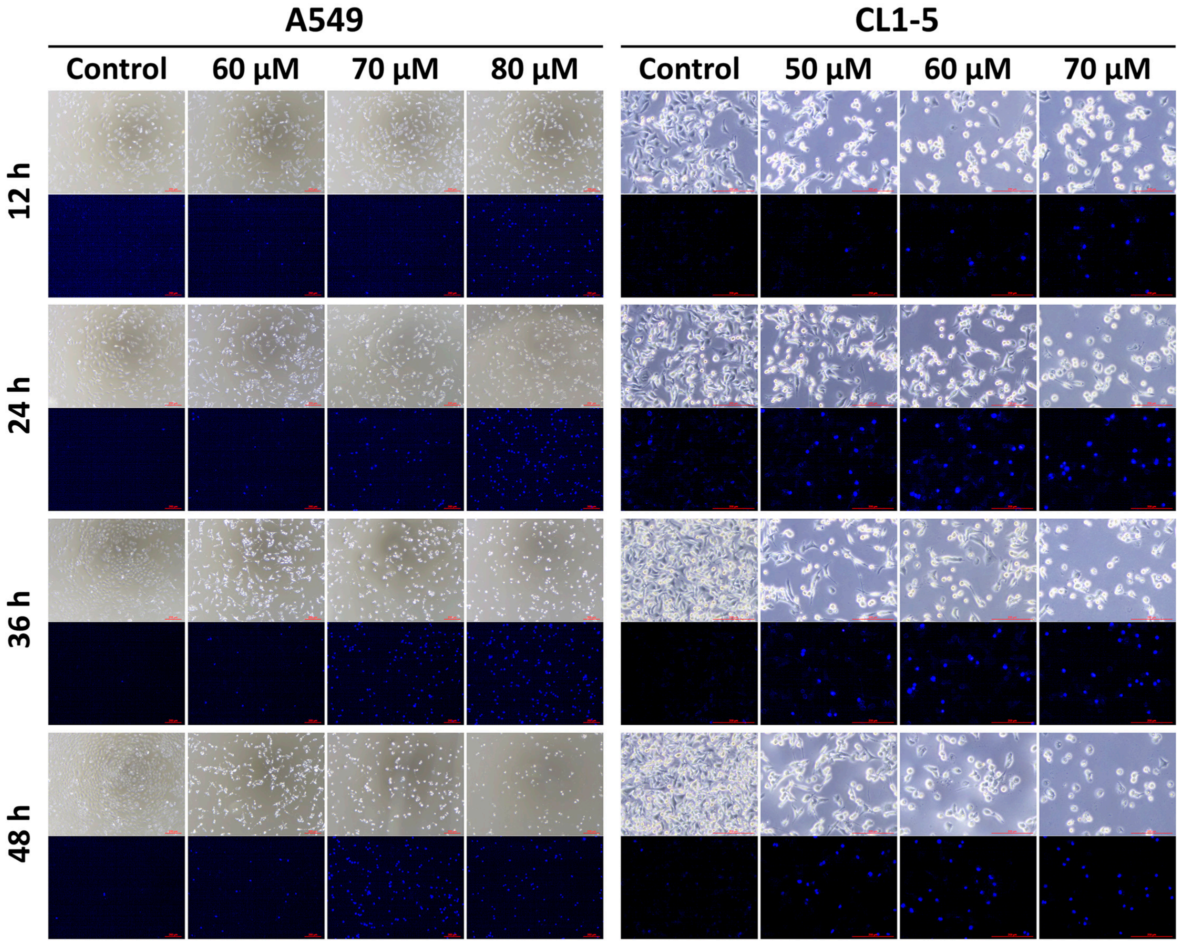


**Figure S1.** Montelukast-induced cell death of lung cancer cells. After being treated with various concentrations of montelukast for the indicated time (12, 24, 36, or 48 h), the cells (A549 and CL1-5) were observed with light microscopy and fluorescence microscopy (DAPI staining).
